# Metallothionein as an Anti-Inflammatory Mediator

**DOI:** 10.1155/2009/101659

**Published:** 2009-05-11

**Authors:** Ken-ichiro Inoue, Hirohisa Takano, Akinori Shimada, Masahiko Satoh

**Affiliations:** ^1^Environmental Health Sciences Division, National Institute for Environmental Studies, 16-2 Onogawa, Tsukuba 305-8506, Japan; ^2^Department of Veterinary Pathology, Faculty of Agriculture, Tottori University, 101 Kozan-cho, Tottori 680-8553, Japan; ^3^Department of Hygienics, Aichi Pharmaceutical University, 1-100 Kusumoto-cho, Chikusa-ku, Nagoya 464-0037, Japan

## Abstract

The integration of knowledge concerning the regulation of MT, a highly conserved, low molecular weight, cystein-rich metalloprotein, on its proposed functions is necessary to clarify how MT affects cellular processes. MT expression is induced/enhanced in various tissues by a number of physiological mediators. The cellular accumulation of MT depends on the availability of cellular zinc derived from the diet. MT modulates the binding and exchange/transport of heavy metals such as zinc, cadmium, or copper under physiological conditions and cytoprotection from their toxicities, and the release of gaseous mediators such as hydroxyl radicals or nitric oxide. In addition, MT reportedly affects a number of cellular processes, such as gene expression, apoptosis, proliferation, and differentiation. Given the genetic approach, the apparently healthy status of MT-deficient mice argues against an essential biological role for MT; however, this molecule may be critical in cells/tissues/organs in times of stress, since MT expression is also evoked/enhanced by various stresses. In particular, because metallothionein (MT) is induced by inflammatory stress, its roles in inflammation are implied. Also, MT expression in various organs/tissues can be enhanced by inflammatory stimuli, implicating in inflammatory diseases. In this paper, we review the role of MT of various inflammatory conditions.

## 1. Introduction

Metallothioneins (MTs) discovered as cadmium-binding protein from horse kidney approximately five decades ago, and were later characterized as a low molecular weight protein with a high cysteine content and a high affinity for divalent important metals, such as zinc and copper, and unimportant ones, such as cadmium and mercury (Margoshes) [1]. Because of their high metal content and unusual bioinorganic structure, they are classified as metalloproteins [2]. MTs are unusually rich in cysteine residues that coordinate multiple zinc and copper atoms under physiological conditions.

## 2. Classification

In mice, there are 4 MT genes that reside in a 50-kb region on chromosome 8 [3]. The mouse MT-I and–II genes are expressed at all stages of development in many cell types of most organs; they are coordinately regulated by metals, glucocorticoids, and inflammatory stress [4]. MT-III is expressed predominantly in not only neurons but also in glia and male reproductive organs [5–7]. MT-IV is expressed in differentiating stratified squamous epithelial cells [3]. All four MT genes are expressed in the maternal deciduum [8]. In humans, whereas, MTs are encoded by a family of genes consisting of 10 functional MT isoforms, and the encoded proteins are conventionally subdivided into 4 groups: MT-1, MT-2, MT-3, and MT-4 proteins [9]. While a single MT-2A gene encodes MT-2 protein, MT-1 protein comprises many subtypes encoded by a set of MT-1 genes (MT-1A, MT-1B, MT-1E, MT-1F, MT-1G, MT-1H, and MT-1X), accounting for the microheterogeneity of the MT-1 protein [2]. As shown above, there are multiple MT genes, expressed in distinct patterns, suggesting that they possess important functions; however, whether they have redundant or divergent functions under both physiological and pathological conditions is not fully understood, although the known functions of MTs include metalloregulatory roles in cell growth, differentiation, and apoptosis and the enhanced synthesis of MTs in rapidly proliferating tissues, implying their crucial role in normal and neoplastic cell growth [10].

## 3. Characteristics

These intracellular proteins are characterized by their unusually high cysteine content (30%) and lack of aromatic amino acids. Because of their thiol rich content, MTs can bind to a number of trace metals such as cadmium, mercury, platinum, and silver, and protect cells and tissues against the toxicity of these metals. Furthermore, MTs are among the most abundant components interacting with the biologically essential metals zinc and copper. MT metal-thiolate fractions, being dynamic and of a high affinity, also facilitate metal exchange in tissues [11].

MTs are present in a great variety of eukaryotes [12], functioning as antioxidants; they also play a protective role against hydroxyl-free radicals. This is relevant in tumors which are known to be markedly radiosensitive, where radiotherapy is the treatment of choice [13].

## 4. Function under Physiological Conditions

The putative functions of MT include intracellular metal metabolism and/or storage, metal donation to target apometalloproteins (especially zinc finger proteins and enzymes), metal detoxification, and protection against oxidants and electrophils [14]. Evidence for these functions originally came from traditional animal, cell culture, and in vitro models. Furthermore, these studies have been supported by experiments using murine models with the targeted deletion or transgenic overexpression of MT genes. MT most likely functions in the regulation of zinc metabolism [14]. Elevations of dietary zinc induce/enhance intestinal MT [15], whereas maximal intestinal Zn accumulation seems to depend on MT synthesis [16]. MT (−/−) mice accumulate less zinc in the distal gastrointestinal tract when fed a high zinc diet [17]. In most studies, zinc absorption was inversely related to the intestinal MT content after MT was induced by dietary, parenteral zinc, or by fasting [14]. Studies using transgenic and knockout mice have confirmed that MT can alter the processing of zinc taken orally because the serum zinc concentration was inversely related to the intestinal MT level in these mice after single oral doses of zinc [17, 18]. In turn, urinary Zn excretion levels measured during a fast or Zn intake restriction were greater in MT (−/−) mice than in MT (+/+) mice [19]. As well, the increase in hepatic Zn concentration after the administration of lipopolysaccharide (LPS) was found in MT (+/+) but not in MT (−/−) mice [20]. These results suggest that MT has the ability to retain Zn under physiological and pathological conditions. On the other hand, tissue Zn concentration was reduced, and the sensitivity to Zn deficiency during pregnancy was enhanced in MT (−/−) mice [21], and further, Zn deficiency caused abnormalities in neonate kidney differentiation in MT (−/−) mice [22]. Conversely, in MT-transgenic mice, Zn was accumulated in female organs, and the teratogenicity of Zn deficiency during pregnancy was significantly ameliorated. Taken together, MT is likely to have a Zn metabolizing activity in the individual level [23].

Besides, MT demonstrates strong antioxidant properties. MT protein levels in rodent liver [24, 25] and mRNA levels in hepatic cell lines [26] are increased following injection with compounds that result in free radical formation, for example, carbon tetrachloride, menadione, or paraquat. An injection of ferric nitrilotriacetate, which produces reactive oxygen species (ROS), induces transcriptional level of MT in the liver and kidney [27]. These findings suggest that MT plays a role in oxidative stress. Consistent with this, MT is able to scavenge a wide range of ROS including superoxide, hydrogen peroxide, hydroxyl radicals, and nitric oxide [19, 28, 29]. In particular, it has been shown that the ability of MT to capture hydroxyl radicals, which are primarily responsible for the toxicity of ROS, is three hundred-times greater than that of glutathione [30], the most abundant antioxidant in the cytosol [19]. Further, metal-thiolate clusters are reportedly oxidized in vitro; thus, they could scavenge deleterious oxygen radicals. Compelling genetic evidence for this concept comes from work using yeast. In brief, yeast that cannot synthesize copper MTs are more sensitive to oxidative stress if they also lack superoxide dismutase, suggesting that yeast MT has antioxidant functions [31]. In addition, the expression of monkey MTs under the control of the yeast MT promoter also protects against oxidative stress [31]. Many agents that induce oxidative stress, such as chloroform, turpentine, diethyl maleate, paraquat, and H_2_O_2_, can also induce MT-I and MT-II in vitro and in vivo [24, 26, 32]. This strongly suggests that MT is involved in protecting against oxidative damage. Conversely, mammalian cells that express excess MTs appear to be resistant to the toxic effects of nitric oxide [33] and many electrophilic antineoplastic agents [34], which are capable of reacting with the cysteines of MT. Further, relatively recent studies have demonstrated that MT is induced by oxidative stress-producing chemicals [35], and exhibits cytoprotection against oxidative stress-related organ damage in vivo [36, 37].

Despite the confirmed roles of MT under physical conditions, as mentioned above, a complete identification of all the functions of this unique protein within an integrative context has yet to emerge, particularly under pathophysiological conditions. Particularly, since proinflammatory cytokines including interleukin (IL)-1, IL-6, and interferon-*γ* also induce hepatic MT gene expression in vivo [38–40], the roles of MT in inflammation have been focused on. There are conflicting reports about the role of MT in inflammatory processes. In fact, MT (−/−) mice were resistant to tumor necrosis factor (TNF)-induced lethal shock compared to MT (+/+) mice [38]. MT-*I*-overexpressing mice are more sensitive to the lethal effects of TNF than MT (+/+) mice [38]. In contrast, Kimura et al. have reported that MT (−/−) mice are more susceptible to LPS-induced lethal shock in D-galactosamine (GalN)-sensitized mice through the reduction of alpha (1)-acid glycoprotein than MT (+/+) mice [41]. Accordingly, it seems that the roles of MT in inflammation depend on pathophysiologic conditions (site, route of stimuli, and type).

Whereas, to date, inflammatory diseases such as systemic inflammatory response syndrome including acute lung injury, allergic asthma, oxidative lung injury, and acute liver injury are as yet refractory and/or hindering daily life, possibly due to the incomplete understanding of molecular targets. Thus, investigation for the role of MT in these inflammatory diseases may provide hint for novel therapeutic options.

## 5. Function of MT under Pathophysiological Conditions in Inflammation

### 5.1. Role of MT in Lung Injury Related to LPS

Previous as well as our recent studies have shown the expression of MT in the lung [39, 42]. Immunohistopathological examination led to the detection of immunoreactive MT-*I/II* proteins in the lungs in endothelial and alveolar epithelial cells of MT (+/+) mice, whereas they were not detected in those of MT (−/−) mice. Furthermore, the expression was confirmed to be enhanced by oxidative stimuli like LPS and ozone (O_3_) exposure (data not shown).

The intratracheal instillation of LPS produces a well-recognized model of acute lung injury, leading to the activation of alveolar macroghages, tissue infiltration of neutrophils, and interstitial edema [43]. Although the inhalation of LPS has been reported to induce MT expression in the lung in vivo [39, 42], there is no evidence regarding the direct contribution of MT in acute lung injury related to LPS.

MT (−/−) and MT (+/+) mice were administered vehicle or LPS (125 *μ*g/kg) intratracheally. Thereafter, the cellular profile of the bronchoalveolar lavage (BAL) fluid, pulmonary edema, lung histology, expression of proinflammatory molecules, and nuclear localization of nuclear factor-*κ* B (NF-*κ* B) in the lung were evaluated. As a result, MT (−/−) mice were more susceptible than MT (+/+) mice to neutrophilic lung inflammation and lung edema, which was induced by intratracheal challenge with LPS. After LPS challenge, MT deficiency enhanced the vacuolar degeneration of pulmonary endothelial and type I alveolar epithelial cells, and caused a focal loss of the basement membrane. However, unexpectedly, LPS treatment induced no significant differences neither in the enhanced expression of proinflammatory cytokines and chemokines, nor in the activation of the NF-*κ* B pathway in the lung between the two genotypes. Lipid peroxide levels in the lungs were significantly higher in LPS-treated MT (−/−) than LPS-treated MT (+/+) mice. These findings suggest that MT protects against acute lung injury related to LPS. The effects are possibly mediated via the enhancement of pulmonary endothelial and epithelial integrity, not via inhibition of the NF-*κ* B pathway [44].

**Figure 1:**
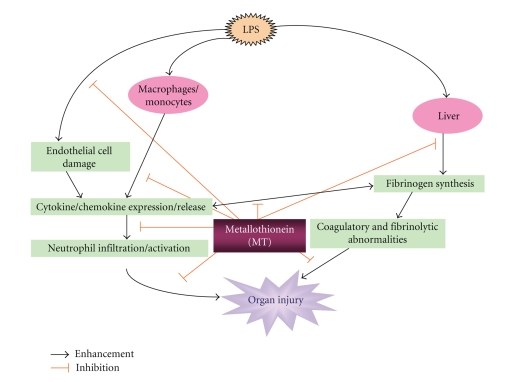
Hypothesized mechanisms of cytoprotection of MT in LPS-related inflammation. Figure reproduced with some modifications with permission from FASEB journal [45].

Next, MT (−/−) and MT (+/+) mice were administered vehicle or LPS (30 mg/kg) intraperitoneally. Thereafter, coagulatory parameters, organ histology (lung, liver, and kidney), and the local expression of proinflammatory molecules were evaluated. As a result, compared with MT (+/+) mice, MT (−/−) mice showed a significant prolongation of the prothrombin time (PT) and activated partial thromboplastin time (APTT), a significant increase in the levels of fibrinogen and fibrinogen/fibrin degradation products, and a significant decrease in activated protein C, after LPS treatment. LPS induced inflammatory organ damage in the lung, kidney, and liver in both genotypes of mice. The damage including neutrophil infiltration in the organs was more prominent in MT (−/−) than MT (+/+) mice after LPS treatment. In both genotypes of mice, LPS enhanced the protein expression of interleukin (IL)-1*β*, IL-6, granulocyte/macrophage-colony-stimulating factor, macrophage inflammatory protein (MIP)-1*α*, MIP-2, macrophage chemoattractant protein-1, and keratinocyte-derived chemoattractant (KC) in the lung, kidney, and liver and circulatory levels of IL-1*β*, IL-6, MIP-2, and KC. In overall trends, however, the levels of these proinflammatory proteins were greater in MT (−/−) than in MT (+/+) mice after LPS challenge. Our results suggest that MT protects against coagulatory and fibrinolytic disturbance and multiple organ damage including lung injury induced by LPS, at least partly, via inhibition of the local expression of proinflammatory proteins in this model (Figure 1) [45]. Nonetheless, its underlying mechanistic pathways including new ones (e.g., Toll-like receptors [46], NALP inflammasomes [47], neurotensin [48], RANK-RANKL [49]) remain to be explored in future.

### 5.2. Role of MT in Allergic Inflammation

Bronchial asthma is a complex syndrome, characterized by obstruction, hyperresponsiveness, and persistent inflammation of the airways. Inflammation in asthma is characterized by an accumulation of eosinophils, lymphocytes, and neutrophils in the bronchial wall and lumen [50–52]. The mechanisms via which inflammatory cells alter airway function in asthmatic conditions include the release of Th2 cytokines (IL-4, IL-5, and IL-13) and chemotactic mediators such as thymus and activation-regulated chemokine, macrophage-derived chemokine, and eotaxin, and various proteases as well as the generation of reactive oxygen species. Thus, next, we determined the role of MT in allergic airway inflammation induced by ovalbumin (OVA) using MT (−/−) mice. MT (−/−) and MT (+/+) mice were intratracheally challenged with OVA (1 *μ*g/body) biweekly 3 times. Thereafter, the cellular profile of the BAL fluid, lung histology, and expression of proinflammatory molecules in the lung were evaluated. After the final OVA challenge, significant increases were noted in the numbers of total cells, eosinophils, and neutrophils in BAL fluid in MT (−/−) mice compared to those in MT (+/+) mice. Histopathologically, in the presence of OVA, the number of inflammatory cells including eosinophils and neutrophils in the lung was larger in MT (−/−) than in MT (+/+) mice. The protein level of IL-1*β* was significantly greater in MT (−/−) than in MT (+/+) mice after OVA challenge. Immunohistochemistry showed that the formations of 8-hydroxy-2′-deoxyguanosine, a proper marker of oxidative DNA damage, and nitrotyrosine in the lung were more intense in MT (−/−) than in MT (+/+) mice after OVA challenge. These results indicate that endogenous MT protects against allergic airway inflammation induced by OVA, at least partly, via suppression of the enhanced lung expression of IL-1*β* and via its antioxidative potential [53].

### 5.3. Role of MT in Oxidative Lung Injury

Ozone (O_3_) is a highly toxic principal oxidant found in urban environments throughout the world. Experimental research has shown that O_3_ inhalation causes airway inflammation/injury in vivo [54]. Furthermore, O_3_ is a strong oxidizing agent that can be rapidly converted into a number of ROS, including hydrogen peroxide [55, 56]. In fact, O_3_-induced lung inflammation/injury comprises oxidative stress-related tissue injury [57–59]. Also, O_3_ exposure reportedly results in oxidative stress in the airway, possibly through the disruption of iron homeostasis [59]; iron can increase oxidant generation after O_3_ interaction with aqueous media and produce hydroxyl radicals [60, 61]. On the other hand, lung expression of MT is reportedly induced by O_3_ exposure in vivo [62, 63]. Thus, we next examined the role of MT in lung inflammation induced by subacute exposure to O_3_ using MT (−/−) mice. After subacute exposure to O_3_ (0.3 ppm), the cellular profile of BAL fluid, pulmonary edema, lung histology, and expression of proinflammatory molecules in the lung were evaluated. Exposure to O_3_ induced lung inflammation and enhanced vascular permeability, which was significantly greater in MT (−/−) than in MT (+/+) mice. Electron microscopically, O_3_ exposure induced the vacuolar degeneration of pulmonary endothelial and epithelial cells, and interstitial edema with focal loss of the basement membrane, which was more prominent in MT (−/−) than in MT (+/+) mice. O_3_-induced lung expression of IL-6 was significantly greater in MT (−/−) than in MT (+/+) mice; however, lung expression of the chemokines such as eotaxin, macrophage chemoattractant protein-1, and keratinocyte-derived chemoattractant was comparable between both genotypes of mice in the presence of O_3_. Following O_3_ exposure, the formation of oxidative stress-related molecules/adducts, such as heme oxygenase-1, inducible nitric oxide synthase, 8-OHdG, and nitrotyrosine in the lung was significantly greater in MT (−/−) than in MT (+/+) mice. Collectively, MT protects against O_3_-induced lung inflammation, at least partly, via the regulation of pulmonary endothelial and epithelial integrity and its antioxidative property (64).

### 5.4. Role of MT in Lethal Liver Injury

Liver has high levels of Zn- and Cu-bound MT and has a high capacity to regenerate. MT is reportedly involved in hepatocyte regeneration after partial hepatectomy [64–67] and chemical injury [68]. Similarly, previous studies have shown that induction of MT can protect animals from hepatotoxicity of several chemicals, such as ethanol, carbon tetrachloride, acetaminophen, and cadmium [69].

Hepatic dysfunction due to liver disorders such as viral hepatitis, liver cirrhosis, and hepatocellular carcinoma is frequently associated with lethal coagulopathy such as DIC. Kimura et al. previously reported that MT is protective against acute liver injury induced by LPS/D- GalN through the suppression of TNF-*α* production/release using MT (−/−) mice [41]. An animal model of acute (lethal) liver injury using LPS/D-GalN develops severe coagulopathy with histological evidence of DIC [70] quite similar to that in humans. Furthermore, most coagulatory factors as well as MT are produced mainly in the liver, indicating a possible role of MT in the pathogenesis of hepatic disorder-related coagulopathy. Besides, our above mentioned study has implicated MT in pathophysiology of coagulatory disturbance [45]. To expand the findings by Kimura et al., therefore, we explored the role of MT in coagulatory disturbance during acute liver injury induced by LPS/D-GalN. Both MT (−/−) and MT (+/+) mice were injected intraperitoneally with 30 *μ*g/kg of LPS and 800 mg/kg of D-GalN dissolved in vehicle. Five hours after the injection, blood samples were collected and platelet counts and coagulatory parameters were measured. LPS/D-GalN challenge significantly decreased platelet number in both genotypes of mice in a time-dependent fashion as compared to vehicle challenge. However, in the presence of LPS/D-GalN, the decrease was significantly greater in MT (−/−) than in MT (+/+) mice. LPS/D-GalN challenge caused prolongation of the plasma coagulatory parameters such as PT and APTT in both genotypes of mice as compared with vehicle challenge. In the presence of LPS/D-GalN, PT and APTT were longer in MT (−/−) than in MT (+/+) mice. The level of fibrinogen significantly decreased 5 hours after LPS/D-GalN challenge in both genotypes of mice as compared to vehicle challenge. After LPS/D-GalN challenge, the level was significantly lower in MT (−/−) than in MT (+/+) mice. As compared to vehicle administration, LPS/D-GalN administration elicited an increase in the plasma level of von Willebrand factor in both genotypes of mice. Further, in the presence of LPS/D-GalN, the level was significantly greater in MT (−/−) than in MT (+/+) mice ([71].

## 6. Conclusion

MTs play important roles in the physiological condition, such as heavy metal homeostasis and radical scavenging. Furthermore, through a genetic approach, MT has been shown to protect against various types of (including LPS-related, allergic, and oxidative) inflammatory conditions in mice, implicating MT-induction/enhancement and/or zinc supplementation to induce/enhance MT as possible therapeutic options for inflammatory diseases, although additional research is needed to conclude its clinical utility.
